# Cognitive perspectives on maintaining physicians’ medical expertise: IV. Best practices and open questions in using testing to enhance learning and retention

**DOI:** 10.1186/s41235-023-00508-8

**Published:** 2023-08-08

**Authors:** Scott H. Fraundorf, Zachary A. Caddick, Timothy J. Nokes-Malach, Benjamin M. Rottman

**Affiliations:** 1 Learning Research and Development Center, University of Pittsburgh, 3420 Forbes Ave., Pittsburgh, PA 15260 USA; 2 Department of Psychology, University of Pittsburgh, Pittsburgh, USA

**Keywords:** Medical expertise, Testing effect, Feedback, Interleaving

## Abstract

Although tests and assessments—such as those used to maintain a physician’s Board certification—are often viewed merely as tools for decision-making about one’s performance level, strong evidence now indicates that the experience of being tested is a powerful learning experience in its own right: The act of retrieving targeted information from memory strengthens the ability to use it again in the future, known as the testing effect. We review meta-analytic evidence for the learning benefits of testing, including in the domain of medicine, and discuss theoretical accounts of its mechanism(s). We also review key moderators—including the timing, frequency, order, and format of testing and the content of feedback—and what they indicate about how to most effectively use testing for learning. We also identify open questions for the optimal use of testing, such as the timing of feedback and the sequencing of complex knowledge domains. Lastly, we consider how to facilitate adoption of this powerful study strategy by physicians and other learners.

## Significance statement

In recent years, there has been a growing call for a greater reliance upon testing as a studying and learning tool for students in the health professions. Indeed, physicians already complete some form of periodic testing in the form of longitudinal assessment for continuing certification. We present evidence that this call is justified insofar as there is robust evidence that the experience of testing can itself be a way to enhance learning and retention. We also discuss what cognitive research implies about how to optimally leverage testing, including longitudinal assessment, as a learning device. Lastly, we discuss how the use case of longitudinal assessment highlights open empirical and theoretical questions regarding the testing effect.

## Introduction

Physicians and other healthcare professionals are tasked with acquiring and maintaining multiple forms of knowledge and cognitive skills, including diagnosis, treatment and management, clinical procedures, interpersonal skills, and basic biological and anatomical knowledge. In recent years, there has been a growing call for a greater reliance upon testing as a studying and learning tool in the health professions (Brown, 2017, EL: 6; Cilliers, 2015, EL: 6; Chesluk et al., 2019; Fung et al., 2019, EL: 6; Griffith et al., 2017; EL: 6; Kulasegaram & Rangachari, 2018, EL: 3; Piza et al., 2019; EL: 5; Rapp et al., 2014, EL: 6; Richmond et al. 2019, EL: 6). These calls typically promote testing in regularly spaced intervals in contrast to “cramming” study behavior (an issue we discuss in further detail below); the combination of testing and spacing over time has been termed *spaced repetition*. Systematic review (Phillips et al., 2019: EL 2) provides evidence that spaced repetition enhances practicing clinicians’ acquisition of knowledge and their clinical behaviors.

Such spaced repetition could be incorporated into the longitudinal assessment programs used in many medical professions. For instance, physicians certified by one of the American Board of Medical Specialties (ABMS) must periodically pass an examination to maintain their certification. Historically, these exams have taken the form of a point-in-time, multiple-choice assessment every six to ten years. More recently, all 24 Boards have announced programs that involve a shift toward more frequent, lower-stakes assessments and test formats that focus on reasoning rather than rote memorization (for further review, see Rottman et al., 2022). One of the primary motivations for this switch is so that these more frequent lower-stakes tests can serve as learning opportunities, rather than just assessment; unlike the older tests, the new longitudinal assessments provide physicians with feedback to promote learning.

In this paper, we examine how such testing can be used to enhance learning and retention of medical expertise. We review the extensive literature on the cognitive benefits of testing on learning and retention. We describe the overall phenomenon as well as how it may be moderated by a number of variables—a key one being feedback—and that may thus constitute best practices for using testing. We consider theoretical explanations for the cognitive mechanisms that underlie the benefits of testing as well whether learners can be trained to employ this helpful learning strategy on their own. Lastly, we consider open questions and future directions in test-enhanced learning. We focus on these principles as they pertain to physicians, as part of a broader collection of five articles in this special issue focused on how physicians maintain medical expertise across their careers, but many of the principles we discuss would also be applicable to maintaining expertise among other healthcare professionals, such as nurses, dentists, or therapists.

This work takes the approach of a narrative review, not systematic, because it covers a wide variety of topics. To situate the strength of the evidence and claims made, we attach evidence levels (EL) to in-text citations for empirical claims (see Table 1). Evidence levels range from 1 to 6, with 1 being the strongest evidence (meta-analyses) and 6 being the weakest (opinion papers).

**Table 1:** Evidence levels for in-text citations for empirical claims

Evidence level	Type of work
1	Quantitative meta-analysis
2	Narrative review
3	Multiple original experiments/randomized controlled trials (RCTs)
4	Single original experiment/RCT
5	Correlational or quasi-experimental study
6	Opinion paper

## Overview and basic design

**Fig. 1:**
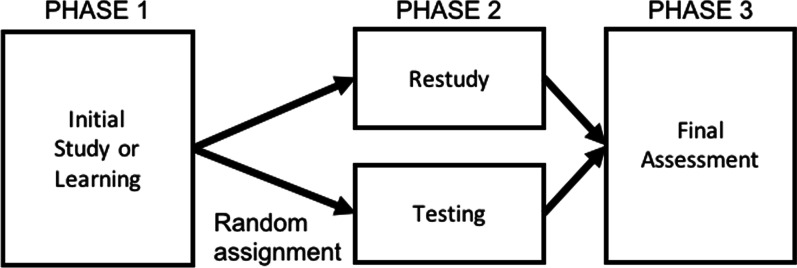
Schematic design of the typical testing-effect study procedure

For over 100 years, psychologists have been aware of the learning benefits of testing one’s own knowledge, including the earliest psychological studies on memory (Abott, 1909; EL: 4; Ebbinghaus, 1885, EL: 5). The basic testing-effect experiment compares, at a minimum, two groups to which individuals are randomly assigned: a restudy group and a testing group (e.g., Carpenter et al., 2008, EL: 4; Karpicke & Roediger, 2008, EL: 4; Roediger & Karpicke, 2006a, EL: 3, 2006b, EL: 3). The restudy group initially studies information and then has an additional study opportunity later. The testing group initially studies information and, instead of restudying the material, is tested on it. (Some experiments also include a third, control group that only initially studies information, e.g., LaPorte & Voss, 1975, EL: 4). The two groups then complete some assessment of memory or performance (see Fig. 1). Critically, by comparing testing to restudying for the same period of time, this design controls for the total time that each group spends engaging with the subject matter; as a result, any differences that emerge are driven by testing itself and not by mere re-exposure to information.

Meta-analytic reviews (Adesope et al., 2017, EL: 1; Rowland, 2014, EL: 1; Yang et al., 2021: EL 1) provide evidence for the benefits of testing over restudy for long-term retention. This phenomenon is often referred to as *the testing effect*, although it has also been referred to as *test-enhanced learning*, *retrieval practice*, and *retrieval-based learning*. The testing effect holds across a wide variety of authentic educational domains (Yang et al., 2021: EL 1), including the natural sciences (Agarwal et al., 2012, EL: 3; McDaniel et al., 2011, EL: 3; McDermott et al., 2014, EL: 3), mathematics and statistics (Hopkins et al., 2016, EL: 4; Kang et al., 2011a, 2011b, EL: 4; Lyle & Crawford, 2011, EL: 4), geography and maps (Carpenter & Pashler, 2007, EL: 4; Rohrer et al., 2010, EL: 3), psychology (McDaniel et al., 2007, EL: 4; Wiklund-Hörnqvist et al., 2014, EL: 4), and history (Agarwal et al., 2012, EL: 4; Carpenter et al., 2009, EL: 4; McDermott et al., 2014, EL: 3; Nungester & Duchastel, 1982, EL: 4; Roediger et al., 2011, EL: 3). Most critically for our purposes, several experiments have shown benefits of retrieval practice for learning among medical students (LaDisa & Biesboer, 2017: EL 5; Raupach et al., 2016: EL 3) and medical residents (Larsen et al., 2009, EL: 4).

How beneficial is testing? Rowland’s (2014, EL: 1) meta-analysis estimated the size of the testing effect as Hedges’ *g* = 0.50; in other words, people randomly assigned to testing scored half a standard deviation (0.50) better than those assigned to restudy, constituting a medium effect size. Adesope et al., (2017: EL 1)’s more recent meta-analysis found an even larger Hedges’ *g* of 0.70. Further, retrieval practice better enhances long-term retention and comprehension than some other popular educational techniques, such as concept mapping (Karpicke & Blunt, 2011, EL: 3). A general conclusion, then, is that being tested is likely to be an effective way of enhancing physicians’ long-term retention of medical expertise.

## Moderators

Researchers have also varied the parameters of the basic testing-effect design presented in Fig. 1 to explore potential moderators of the testing effect, which we now review.

### Retention interval and “cramming”

One important characteristic of any learning task is the *retention interval*—the time between initial learning (e.g., reading a document or taking a practice test) and the final assessment. The benefits of testing for retention remain even when assessed 8 to 24 months later (Agarwal et al., 2012, EL: 4; Kerfoot, 2009, EL: 4). In fact, the benefits of testing relative to restudy are intensified with a longer retention interval, a phenomenon known as the *test-delay interaction* (e.g., Agarwal et al., 2012, EL: 4; Chan, 2010, EL: 4; Roediger & Karpicke, 2006a, EL: 3; Rowland, 2014, EL: 1; Runquist, 1983, EL: 3; Toppino & Cohen, 2009, EL: 3; Wheeler et al., 2003, EL: 3; Yeo & Fazio, 2019, EL: 3). For example, Rowland’s (2014, EL: 1) meta-analysis found that the difference between testing and restudy was larger when the retention interval was longer than a day (Hedges’ *g* = 0.69) than when the retention interval was less than a day (Hedges’ *g* = 0.41). Put another way, testing is particularly beneficial when material must be retained for a long time; although cognitive skills decline on the whole over a longer retention interval (Rubin & Wenzel, 1996, EL: 2; Wixted, 2004, EL: 3), this decline is *smaller* with testing relative to restudy.

However, there is one circumstance in which testing is *not* more beneficial than restudy: when the final test immediately follows practice. Under these circumstances (i.e., “cramming” immediately before a test), restudy outperforms retrieval practice (e.g., Roediger & Karpicke, 2006a, EL: 3; Toppino & Cohen, 2009, EL: 4; Wheeler et al., 2003, EL: 3). In sum, in the very short term, restudy may be better than testing, but testing quickly becomes superior over the long term. Since physicians need to retain information over years if not decades, periodic testing should be more beneficial for retention than mere restudy.

### How much testing: frequency, length, repetition

Given that testing benefits long-term retention, one might ask how much testing we can feasibly ask learners to do: How long should each test be, and is there a point at which additional testing becomes harmful? Some research suggests a *list length effect* whereby, as the amount of material to be learned increases (i.e., a longer practice test), the probability of learning any individual item decreases (Cary & Reder, 2003, EL: 3; Gillund & Shiffrin, 1984, EL: 4; Gronlund & Elam, 1994, EL: 4; Ohrt & Gronlund, 1999, EL: 3; Ratcliff et al., 1990, EL: 4; Strong, 1912, EL: 4). However, others have argued that the list-length effect disappears when various confounders are carefully controlled (Dennis & Humphreys, 2001, EL: 3; Dennis et al., 2008, EL: 3; Kinnell & Dennis, 2011, EL: 3), and, at any rate, the *total* amount learned is greater with longer lists (Murayama et al., 2016, EL: 3; Ward, 2002; EL: 4). In sum, there does not appear to be any *cognitive* reason to avoid longer tests, and this decision can instead be made based on time and motivational constraints.

A related question concerns *how many times* learners should be tested on the same material. The literature suggests that the benefits of multiple tests are nuanced. On the one hand, adding a second test—or even more—does enhance retention above and beyond the first (Roediger & Karpicke, 2006a, 2006b, EL: 3; Karpicke & Roediger, 2007, EL: 4; Pyc & Rawson, 2009, EL: 3; Wheeler & Roediger, 1992, EL: 3; Yang et al., 2021: EL 1). Even if learners answered correctly on the first test, further study can still enhance long-term retention, a strategy known as *overlearning* (e.g., Karpicke & Roediger, 2007, EL: 4; Karpicke, 2009, EL: 3; Kornell & Bjork, 2008, EL: 3; Postman, 1965, EL: 4; Pyc & Rawson, 2011, EL: 4; Rawson & Dunlosky, 2011, EL: 3; Vaughn & Rawson, 2011, EL: 3). Overlearning is thought to benefit retention because it provides further feedback and strengthens memory traces to buffer against future forgetting (Driskell et al., 1992, EL: 1). Relative to the common strategy of dropping items from testing once they have been answered correctly a single time, overlearning has a medium to large benefit on long-term retention, *d* = 0.75 (Driskell et al., 1992, EL: 1). On the other hand, the benefit from the first test is much larger than the additional benefit from a second test (or from a second episode of practice more generally; Dunlosky & Hertzog, 1997, EL: 4; Koriat et al., 2002, EL: 3; Rawson & Dunlosky, 2011, EL: 3; Vaughn & Rawson, 2011, EL: 3; Yang et al., 2021: EL 1), such that additional tests yield diminishing returns. In sum, there is some moderate benefit to continuing to occasionally practice even learned concepts, but many benefits from retrieval practice could be realized with just one test.

### Timing of tests: spaced learning

When should learners be tested? Cognitive scientists have extensively studied the broader question of when to schedule learning, whether in the form of restudying or testing. As we discussed above, practicing twice is somewhat better than practicing once (Madigan, 1969, EL: 4). Critically, a second learning session is particularly beneficial when learning episodes are spaced over time (*distributed practice*) rather than back-to-back (*massed practice*; i.e., cramming), even when controlling for the total amount of time spent studying (Cepeda et al., 2006, EL: 1; Crowder, 1976, EL: 3; Madigan, 1969, EL: 4; c.f., Timer et al., 2020, EL: 4). This effect has been referenced with varying terminology in the literature, including *the spacing effect*, *spaced education*, *spaced training*, and *distributed practice* (Versteeg et al., 2019, EL: 2). For the purposes of the current review, we will use the term *spaced learning*.

Benefits of spaced learning cannot be attributed merely to inattention or boredom with massed study, since spaced learning is still better even when attention is measured and tightly controlled (Zimmerman, 1975, EL: 4). Rather, many contemporary theoretical accounts propose that distributed practice potentiates memory because each subsequent study episode reminds the learner of the previous episode or episodes, re-activating and strengthening them in memory (Benjamin & Tullis, 2010, EL: 4; Bjork & Bjork, 1992, EL: 3; Jacoby & Wahlheim, 2013, EL: 3; McKinley & Benjamin, 2020, EL: 3; Tullis et al., 2014, EL: 3).

Further, even when using spaced learning, spacing study episodes with longer gaps (*lags*) is generally better than spaced learning with relatively short gaps, which has been termed the *lag effect* (Cepeda et al., 2006, EL: 1; Crowder, 1976, EL: 3; Madigan, 1969, EL: 4; Melton, 1967, EL: 4). The spacing and lag effects extend to testing such that, given multiple tests, a longer lag between two tests leads to better retention (Pyc & Rawson, 2009, EL: 3). However, extremely long lags may be harmful (Cepeda et al., 2009, EL: 3; Cepeda et al., 2008, EL: 3). The optimal lag is likely to depend on the retention interval: The longer that learners need to retain what they have learned, the longer the ideal gap in spaced learning (Cepeda et al., 2008, EL: 3). Since physicians generally need to retain their expertise for years if not decades, spacing practice over a long span of time—such as through longitudinal assessment—is likely to result in the most enduring medical knowledge.

Interventions in the health sciences have sometimes combined the testing effect and spaced learning by having learners answer test questions periodically over time, a practice often termed *spaced repetition*. Systematic review (Phillips et al., 2019: EL 2) indicates that spaced repetition enhances healthcare professionals’ acquisition of knowledge and their clinical behaviors (as measured both via self-report and objective records). Further, spaced repetition activities generally meet with acceptance and uptake; in the studies reviewed by Phillips et al., 87% of participants in spaced-repetition interventions indicate they would participate in future spaced-repetition activities, and completion rates were high. Not all of the studies of healthcare professionals reviewed by Phillips et al. (2019) involved physicians (e.g., some involved nurses), and only some used randomized controlled trials with experimental designs, indicating a need for more high-quality studies specifically with physicians. Nevertheless, Phillips et al. (2019) concluded that spaced repetition is “one of the few evidence-based pedagogies that can increase knowledge, promote retention of knowledge […] and positively affect clinical practice” (p. 899).

### Test format and type of knowledge

Physicians are tasked with acquiring and maintaining several different types of knowledge, such as basic factual knowledge, diagnosis and classification, medical procedures and clinical behaviors. Could testing enhance retention of each of these?

In general, testing indeed appears to be effective across many testing formats and types of knowledge. Benefits of testing for retrieval have been demonstrated for most basic memory tasks: *recognition* tasks in which the learner merely identifies a stimulus as previously encountered or not (e.g., multiple-choice or yes/no tests, or deciding whether you recognize a person; Adesope et al., 2017, EL: 1; Rowland, 2014, EL: 1; Yang et al., 2021: EL 1), *cued recall* tasks in which the learner supplies partial information in response to a cue (e.g., a fill-in-the-blank test, or answering a question asked by a patient; Adesope et al., 2017, EL: 1; c.f., Hinze & Wiley, 2011, EL: 4; Rowland, 2014, EL: 1), and *free recall* tasks in which the learner must bring to mind information without any guide from the environment (e.g., an essay test; Adesope et al., 2017 EL: 1; Hinze & Wiley, 2011, EL: 4; Rowland, 2014, EL: 1). Adesope et al., (2017, EL: 1) formally examined test format in their meta-analysis and found a significant benefit of testing over restudy for all test formats. For this reason, the specific format of a test item is likely of less importance than the presentational quality of the question (e.g., clarity, readability, and veracity of text).

Some controversy has existed as to whether testing benefits more complex knowledge types and tasks, such as problem-solving (c.f., Karpicke & Aue, 2015, EL: 6; Leahy et al., 2015, EL: 4; Rawson, 2015, EL: 2; van Gog & Kester, 2012, EL: 4; van Gog et al., 2015, EL: 4; van Gog & Sweller, 2015, EL: 3). However, meta-analytic evidence suggests testing does benefit complex problem-solving tasks and other types of high-level conceptual knowledge (Yang et al., 2021: EL 1), and several studies have found benefits of testing specifically for clinical behaviors and skills (Kromann et al., 2009, EL: 4; Larsen et al., 2009, EL: 4; Raupach et al., 2016, EL: 3). Another finding relevant to medical expertise is that testing benefits laboratory *classification* tasks, such as learning to classify different families of birds based on individual photo exemplars (Jacoby et al., 2010, EL: 4; Siler & Benjamin, 2019, EL: 3), somewhat analogous to diagnosing or classifying patients.

In sum, the testing effect appears to play out for many different formats and types of knowledge—including those relevant to longitudinal medical expertise, such as classification, medical procedures, and the basic formats used in standard computerized testing.

### Ordering of practice material

Given that the content to longitudinal assessments generally includes multiple concepts and items, a natural question is whether there are better or worse ways to order such material. The optimal ordering of learning material has frequently studied in cognitive psychology, although not always in the specific context of the testing effect. Cognitive psychologists who have studied this issue more broadly have often contrasted two extremes of scheduling material for practice. We follow Brunmair and Richter (2019) by defining a *blocked* schedule as one in which *all* problems or examples pertaining to one topic are presented before moving on to the next topic or concept—similar to the organization of most textbooks or courses in formal education. For instance, a physician may study many examples of hyperthyroidism, then many examples of diabetes. By comparison, an *interleaved* schedule is defined as any ordering in which the to-be-practiced concepts are intermixed such that examples of one category are not fully exhausted before moving onto the next. For example, a physician may review some hyperthyroidism cases and some diabetes cases mixed together (in any order), rather than grouped by diagnosis. Meta-analysis (Brunmair & Richter, 2019, EL: 1; Firth et al., 2021, EL: 1) suggests that, for most materials, interleaving practice results in superior learning than blocked practice, with a medium effect size.

Of course, various intermediate schedules are also possible, such as beginning with blocked practice and then transitioning to interleaved (Yan et al., 2017, EL: 5). Preliminary evidence suggests that an intermediate degree of interleaving is optimal in more complex domains, such as when topics are arranged a hierarchical structure at multiple levels of organization (Yan & Sana, 2021: EL 4) or when individual items can be cross-classified in multiple topics (Abel et al., 2021: EL 3).

One reason that interleaving is thought to benefit learning is that it calls attention to the *differences* between concepts (Brunmair & Richter, 2019, EL: 1; Carvalho & Goldstone, 2015, EL: 3; Carvalho & Goldstone, 2017, EL: 3; Kang & Pashler, 2012, EL: 3). For example, learning to distinguish two potentially confusable patient presentations (e.g., shortness of breath could reflect heart problems or lung problems) requires understanding what the two diagnoses have in common, but especially what differentiates them. Likewise, learning to choose between two treatments that could both be used in a given situation requires understanding why they could both be used, but especially why there is a reason to choose one over the other. Thus, one recommendation is that, when there is a concern that two diagnoses or two treatments may be confused (i.e., they may be subject to interference; Caddick et al., 2022), it would likely be beneficial to interleave those concepts together on the *same* assessment rather than blocked into different assessments. One caveat is that much of the work on the interleaving benefit has not been specific to retrieval practice (e.g., in some studies, learners merely viewed the exemplars without being tested) and it would be useful to confirm the benefits of interleaving specifically in the context of retrieval practice.

One other line of work has explored item sequencing specifically in the context of test items and their difficulty. This work is grounded in the more general principle of the *peak-end rule*: people tend to judge experiences primarily as a function of (a) the affective peak (i.e., the strongest positive or negative experience) and (b) their ending experience (Diener et al., 2001, EL: 3; Do et al., 2008, EL: 3; Kahneman et al., 1993, EL: 4). In line with this, laboratory studies have shown that adding easier items, which are likely to engender a positive experience of success, to the end of a test increases learners’ willingness to engage in future testing, even when the additional items extend the overall length of the test (Cho, 2021, EL: 4; Finn & Miele, 2016, EL: 3; Finn & Miele, 2021, EL: 3; O’Day, 2022, EL: 3). Consequently, we suggest there may be potential value in ending each longitudinal assessment “on a high note” with a few relatively easy items that are likely to encourage continued participation in the program.

### Transfer to untested material

Evidence suggests that retrieval practice can support *transfer*: a benefit to learning not just on the exact tested item, but on related items or material (Carpenter, 2012, EL: 3; Pan & Rickard, 2018, EL: 1; Yang et al., 2021: EL 1). It is generally rare—if not impossible—to observe *far transfer*, where training or practice in one domain also confers benefits to other, wholly unrelated domains (Sala & Gobet, 2017, EL: 1). However, the learning benefits of the testing effect do appear to transfer to more closely related material (*near transfer*). For example, Kang et al., (2007, EL: 4) found that retrieval practice transfers between test formats: College students who practiced in the form of multiple-choice questions also showed benefits on a final short-answer test (relative to restudy or no-review conditions), and vice versa (see also Lyle & Crawford, 2011, EL: 4).

Retrieval practice can sometimes also transfer from the practiced information to other, related information. In a college neuroscience course, McDaniel et al., (2007, EL: 4) presented students with fill-in-the-blank quiz questions, such as *All preganglionic axons, whether sympathetic or parasympathetic, release* ____ *as a neurotransmitter*. Practice on these questions benefited subsequent exam performance even when students were tested on a different piece of information from the same statement, such as *All _____ axons, whether sympathetic or parasympathetic, release acetylcholine as a neurotransmitter*. Similarly, the benefits of being tested on part of a science text can sometimes generalize to other, related facts from the text (Chan, 2010 EL: 4; Chan et al., 2006, EL: 4), though this has not been observed in all studies (Pan & Rickard, 2018, EL: 1; Woolridge et al., 2014, EL: 3).

Finally, retrieval practice can transfer between levels of knowledge or analysis (Agarwal et al., 2013, EL: 4; Butler, 2010, EL: 3; Pan & Rickard, 2018, EL: 1; Rohrer et al., 2010, EL: 3). For example, practicing the notion of *competition* with a definition question (“What is the term for when two or more organisms vie for limited environmental resources?”) also benefits application (e.g., “A group of 500 pandas are living in a reserve. Recent dry weather has reduced the bamboo populations, which the pandas rely on. The pandas are in what type of relationship?”), and vice versa (Agarwal et al., 2013, EL: 4).

These results imply that learners who use testing are not just memorizing the answers to specific test items; they are developing their understanding of the concept more broadly. An implication for longitudinal assessment of medical expertise is that being tested should improve physicians’ retention not just of the specific tested material, but of other, related material as well.

### Individual differences

More recent work has begun to examine whether the testing effect applies equally across groups of learners. Meyer and Logan (2013, EL: 4) found that older adults benefit from testing just as much as college-age learners. This finding is relevant to longitudinal assessment of medical expertise because it suggests that testing may be beneficial even for physicians more advanced in their career and further removed from training.

One question of particular interest is how the testing effect may be modulated by prior knowledge of the tested domain. Several studies have examined whether the degree of learners’ prior knowledge correlates with the magnitude of the testing effect, with mixed results: One study found that retrieval practice has a compensatory effect such that it is more beneficial for learners with low existing topic knowledge (Cogliano et al., 2019, EL: 5), another conversely found that retrieval practice is more beneficial for learners with *high* knowledge (Carpenter et al., 2016, EL: 5), and others found testing equally effective regardless of prior topic knowledge (Glaser & Richter, 2022, EL: 5; Xiaofeng et al., 2016, EL: 5), although all of these studies are limited by their correlational nature. More recently, in an experimental study, Buchin and Mulligan (2023, EL: 4) manipulated learners’ topic knowledge by having them study an academic topic across multiple days of training before introducing a retrieval-practice manipulation; this study found that the testing effect equally benefited high-knowledge and low-knowledge learners.

Other work has examined how the relevance of testing may be modulated by more general academic aptitude or cognitive abilities. The boost provided by testing may be especially helpful for students who would otherwise struggle: A larger testing effect has sometimes been observed for learners lower in the ability to hold information in active memory (*working memory capacity*; Agarwal et al., 2017, EL: 5), in reading comprehension (Callender & McDaniel, 2007, EL: 5), or in general intelligence (Brewer & Unsworth, 2012, EL: 5). Because working memory typically declines with age (Park et al., 2002, EL: 5), this may make testing particularly important for older physicians. However, other studies have found testing benefits to be equal regardless of working memory or general intelligence (Bertilsson et al., 2021, EL: 5; Jonsson et al., 2021, EL: 5; Pan et al., 2015, EL: 5; Wiklund-Hörnqvist et al., 2014, EL: 5).

In general, then, there does not seem to be consistent evidence that retrieval practice benefits only a select group of learners, either in terms of prior knowledge or general cognitive ability. Instead, Jonsson et al. (2021) conclude that retrieval practice is “a learning method for all.” This means that physicians are likely to be among those who benefit from the testing effect, and moreso that testing could help physicians across a range of backgrounds and knowledge.

## Feedback after testing

When learners are tested—either during practice tests or final assessments—most will answer some of the items that they have studied or practiced correctly but make errors on others. One concern sometimes expressed by educators and learners is that these self-generated errors may become (falsely) incorporated into learners’ knowledge base, and so perhaps a more didactic approach that prevents learners from making mistakes would be better (e.g., *errorless learning*; for further discussion, Metcalfe, 2017, EL: 3; Middleton & Schwartz, 2012, EL: 2).

Evidence indicates that the benefits of testing for long-term learning do indeed depend in part on how well learners perform on the test (Rowland, 2014, EL: 1). When no feedback is provided during testing, individuals receive a positive memory boost for correctly recalled information (Kornell et al., 2011, EL: 3; Rowland, 2014, EL: 1; Spellman & Bjork, 1992, EL: 6). However, for the items with weak memory strength that are not correctly recalled on the no-feedback test, no memory boost occurs. In this way, tests without feedback may create an asymmetry or *bifurcation* in learning dependent upon pretest memory strength for individual pieces of information. In contrast, restudy conditions provide a memory boost for all items reviewed, but it is a weaker boost than received for correctly recalled items in the test condition.

However, this asymmetry can be alleviated by the addition of feedback after a retrieval practice attempt. Thus, although testing is beneficial even without feedback, testing *with* feedback is even better (Butler & Roediger, 2008, EL: 4; Rowland, 2014, EL: 1; Yang et al., 2021: EL 1; c.f., Adesope et al., 2017, EL: 1). Indeed, as long as feedback is given, errors generated by learners in practice testing do not impair long-term performance (Butler et al., 2008, EL: 3; Huelser & Metcalfe, 2012, EL: 3; Kang et al., 2011a, 2011b, EL: 3; Kornell et al., 2015, EL: 3; Kornell et al., 2009, EL: 3; Kornell & Metcalfe, 2014, EL: 4; Metcalfe, 2017, EL: 3; Metcalfe & Kornell, 2007, EL: 4; Richland et al., 2009 EL: 3; c.f., Knight et al., 2012, EL: 3, for more mixed results). In fact, testing with feedback is so powerful that an unsuccessful retrieval attempt followed by feedback is more beneficial than simply reading the correct information without attempting retrieval (Kornell et al., 2009, EL: 4; Hays et al., 2013, EL: 4; Richland et al., 2009 EL: 4). Thus, the concern that errors during learning undermine long-term knowledge is unfounded so long as feedback is given.

Further, because corrective feedback allows people to learn even from difficult tests, feedback allows learners to be presented with more challenging and demanding tests (e.g., short answer rather than multiple choice) that lead to better learning (Kang et al., 2007, EL: 3). Thus, training that permits errors can be more effective than errorless learning (Keith & Frese, 2008, EL: 1) because it allows learners to capitalize on testing and practice effects. These findings imply that tests will most benefit physicians’ retention of medical expertise if (a) feedback is given, especially for more difficult material, and (b) tests are appropriately challenging.

### How should feedback be given?

The form of feedback clearly matters: Simply stating whether a response is correct or incorrect (*verification feedback*) confers little or no benefit whereas presenting the actual, correct answer benefits learning (Bangert-Drowns et al., 1991, EL: 1; Fazio et al., 2010, EL: 3; Metcalfe, 2017, EL: 3; Moreno, 2004, EL: 3; Pashler et al., 2005, EL: 4; Whyte et al., 1995, EL: 4) although this may be qualified by the learner’s knowledge level (Hausmann et al., 2013, EL: 5).

Some studies have also examined additional elaborations that can be provided beyond correct-answer feedback. One popular technique is to present an explanation of why the correct answer is correct; however, most studies have found that such *explanatory feedback* does not yield gains over providing the correct answer alone (Bangert et al., 1991, EL: 1; Corral & Carpenter, 2020, EL: 4; Kulhavy et al., 1985, EL: 4; Mandernach, 2005, EL: 4; Smits et al., 2008, EL: 4; Whyte et al., 1995, EL: 4, but see Butler et al., 2013, EL: 3, for somewhat more mixed results). Indeed, providing additional feedback to read may be less efficient overall (Kulhavy et al., 1985, EL: 4). On the other hand, one study suggests that providing *examples* of an incorrectly understood concept can enhance learning beyond presenting the answer alone (Finn et al., 2018, EL: 3), but, to date, there is not much research on this approach. In sum, there is evidence that feedback should include the correct answer, but further explanation beyond that may be unnecessary.

Another relevant feature of feedback is its reliability and validity. Gnepp et al., (2020, EL: 3) found that individuals may be skeptical of negative feedback when the feedback provider’s accuracy or credentials are in question. This study examined workplace feedback from a manager, and it likely differs from the relative objectivity offered by an automated system providing feedback about errors. Still, it suggests there may be value to citing information sources in feedback to add authority and objectivity.

### When should feedback be given?

Some work has also examined the timing of feedback, generally contrasting immediate feedback with feedback that is delayed to some degree. In controlled laboratory studies, feedback delayed by several hours or days is often more effective (Butler & Roediger, 2008, EL: 4; Kulik & Kulik, 1988, EL: 1; Schmidt & Bjork, 1992, EL: 3; Schooler & Anderson, 1990, EL: 4), or at least no worse (Kang et al., 2011a, 2011b, EL: 4; Metcalfe et al., 2009, EL: 4; Smits et al., 2008, EL: 4). Delayed feedback may better potentiate long-term retention and learning because it encourages learners to develop their own monitoring and self-assessment skills, rather than relying exclusively on external feedback (Schmidt et al., 1989, EL: 4). On the other hand, in in vivo classroom studies, the reverse seems to be true: immediate feedback is better than delayed (Kulik & Kulik, 1988, EL: 1; Lemley et al., 2007, EL: 4). This reversal has been attributed to the fact that, in a busy classroom environment, students may not even attend to feedback when it is delayed because their priorities may have since shifted (Kulik & Kulik, 1988, EL: 1; Metcalfe, 2017, EL: 3).

What does this imply for longitudinal assessment of medical expertise? Given that physicians are likely motivated to attend to the feedback they receive, the literature suggests that delayed feedback may be superior, but there is a need to test this specifically within the medical domain. Some evidence does suggest that a particularly effective strategy may be to interleave periods of testing with periods of restudy so that learners can restudy material they answered incorrectly (McDaniel et al., 2015, EL: 4; Metcalfe & Miele, 2014; EL: 4), then incorporate the corrected information into their next retrieval attempt.

### Why does feedback help?

Why is feedback so effective at ameliorating errors? One possible mechanism, of course, is that feedback simply presents another opportunity to encounter correct information. This is supported by the fact that, as we reviewed above, verification feedback alone is not particularly helpful; the correct answer must be provided (Bangert et al., 1991, EL: 1).

Another important factor may be that, when an error is committed with high confidence, the resulting negative feedback can be especially memorable (the *hypercorrection effect*; Butler et al., 2011, EL: 4; Butterfield & Metcalfe, 2001, EL: 5; Butterfield & Metcalfe, 2006, EL: 5; Cyr & Anderson, 2012, EL: 5; Fazio & Marsh, 2009, EL: 5; Fazio & Marsh, 2010, EL: 5; Iwaki et al., 2013, EL: 5; Metcalfe, 2017, EL: 3; Metcalfe & Finn, 2011, EL: 5; Sitzman et al., 2015, EL: 5). The importance of such hypercorrective feedback accords with multiple theoretical perspectives in cognitive science, such as *error-based learning* views, in which learning occurs to the degree that preceding expectations are incorrect (*prediction error*; e.g., Clark, 2013, EL: 3; Dell & Chang, 2014, EL: 3; Rumelhart & McClelland, 1986, EL: 3), and Bayesian views, in which cognition can be viewed as updating a set of beliefs in accordance with the experienced “data” or world (e.g., Frank & Goodman, 2012, EL: 4; Jacobs & Kruschke, 2010, EL: 3; Tenenbaum et al., 2011, EL: 3). Thus, feedback seems particularly effective at alleviating *intrusions—*the false “recall” of incorrect information*—*rather than failures to recall anything at all (Butler & Roediger, 2008, EL: 4). In other words, it is especially important to give feedback when learners respond incorrectly rather than when they decline to respond.

A related phenomenon, converse to the hypercorrection effect, is that if the learner *is* correct, but has low confidence (e.g., a “lucky guess”), feedback increases the probability that this correct response will be retained later (Agarwal et al., 2012, EL: 4; Butler et al., 2008, EL: 3; Fazio et al., 2010, EL: 3; c.f., Pashler et al., 2005, EL: 4). Thus, we recommend providing feedback for correct as well as incorrect responses. Although feedback may be redundant when a learner is highly confident in their response *and* correct, it is unlikely to negatively affect learning (Hays et al., 2010, EL: 4; Karpicke & Roediger, 2008, EL: 4).

Finally, feedback can perhaps serve as a cue to forget or inhibit incorrect information.^1^ In general, when people are explicitly told that some information is incorrect, obsolete, or otherwise should now be forgotten, they can favor retention of other, to-be-remembered information (the phenomenon of *directed forgetting*; MacLeod, 1998; EL: 2; Sahakyan et al., 2013, EL: 2). Feedback that one is incorrect or has performed poorly may be a cue to initiate this directed forgetting process on erroneous knowledge.

## Training people to use retrieval practice

Most research on the testing effect has focused on testing administered by educators and professional organizations. However, learners can also choose to test themselves as a learning strategy. Unfortunately, research indicates that, on the whole, learners use this strategy only rarely; students often prefer less efficacious strategies, like re-reading (Karpicke et al., 2009, EL: 5; Kirk-Johnson et al., 2019, EL: 5), including learners in the health sciences (Coker et al., 2018; EL: 5; Jouhari et al., 2016, EL: 5; Piza et al., 2019, EL: 5). Further, even those who *do* employ testing might do it for other reasons—for instance, to assess what they have learned from other study activities rather than as a learning activity in its own right (Hartwig & Dunlosky, 2012, EL: 5; Kornell & Son, 2009: EL 5).

Nevertheless, some learners *do* use testing to study, and they appear to reap learning benefits from it. In laboratory studies, learners who choose to employ more testing show better retention (Karpicke, 2009, EL: 5). Outside of the laboratory, college students who report using more retrieval practice in their own self-regulated learning have higher GPA (Hartwig & Dunlosky, 2012, EL: 5). This conclusion also extends to medical students: Students who employ more practice testing perform better in the first year of medical study (Baatar et al., 2017, EL: 5; West & Sadoski, 2011, EL: 5) and on medical licensing examinations (Burk-Rafel et al., 2017, EL: 5; Deng et al., 2015, EL: 5); West and Sadoski (2011, EL: 5) and Burk-Rafel et al., (2017, EL: 5) both found that the retrieval practice in self-directed study *better* predicts performance than more general academic measures, such as MCAT scores and undergraduate GPA. Although these studies are correlational, when combined with the experimental evidence for the testing effect discussed above, the role of retrieval practice in these students’ learning is likely causal. In sum, the literature suggests that many learners, including medical students, do not often leverage retrieval practice, but those who do benefit in their knowledge and academic performance.

Why don’t more learners engage in these useful study behaviors? First, they may be aware of the benefits of testing but do not implement it because of the required time and effort and other costs (see also Nokes-Malach et al., 2022). For example, Coker et al., (2018, EL: 5) found that 90% of surveyed pharmacy students believed their learning would benefit from regular retrieval practice, but only 60% engage in it. Second, students may not have been taught beneficial learning strategies to begin with: Piza et al., (2019, EL: 5) found that the majority of the health profession faculty they surveyed held misconceptions about evidence-based study practices.

As a result, some researchers have examined whether learners can be taught to use testing approaches for learning. Some evidence suggests that individuals who have more formal education in cognitive psychology (McCabe, 2011, EL: 5) or who are assigned practice that allows them to experience the testing effect (Ariel & Karpicke, 2017, EL: 4; Einstein et al., 2012, EL: 5; Tullis et al., 2013, EL: 4) come to appreciate the value of testing and incorporate it into future study plans. A workshop specifically designed to teach retrieval practice as a study strategy increased both college students’ intention to apply retrieval practice and their resulting exam performance (Stanger-Hall et al., 2011, EL: 4). And after implementing a supplemental spaced-repetition learning system with attendees at a continuing medical education conference, Shaw et al., (2011, EL: 3) found that 97% of participants stated interest in participating in the system again in the future. An implication for longitudinal assessment of medical expertise, then, is that if physicians are guided to experience the learning benefits of self-testing, they may also adopt more effective study and learning procedures even beyond the assessment itself.

## Mechanisms

Understanding *how* and *why* retrieval practice works is important for applying it across situations: A strong theoretical account of the testing effect generates predictions about when and where it can be used, rather than requiring each new application (e.g., each new test format, subject matter, or group of learners) to be tested afresh. Further, a clear explanation of why retrieval practice works can facilitate outreach to learners and educators.

The testing effect is consistent with several broad principles of human cognition. The benefits of practicing retrieval can be seen as an instance of *transfer-appropriate processing*: The activities that make for the most effective learning are generally those that match the way the material will be used later (Roediger & Blaxton, 1987, EL: 3; Roediger & Butler, 2011, EL: 3). For example, reading the driver’s manual would be ideal practice for taking a written driver’s exam, whereas behind-the-wheel experience would be ideal practice for actually driving. It follows from this principle that the best way to potentiate later retrieval is to practice retrieval itself, rather than to reread or perform other activities less closely related to retrieval. Supporting this account, Adesope et al., (2017, EL: 1; c.f., Rowland, 2014, EL: 1) found evidence in their meta-analysis that similarity of initial and final test moderates the testing effect. When practice tests and final tests use identical test formats, a somewhat larger testing effect occurs (Hedges’ *g* = 0.63), compared to when practice tests and final tests differed in format (Hedges’ *g* = 0.53).

However, the value of testing may not always be obvious to learners (or educators). Although testing facilitates long-term retention, it may require initial processing that is more effortful or less accurate, as learners struggle with practice questions and sometimes answer them erroneously or not at all. Thus, retrieval practice can be viewed as a *desirable difficulty*: the principle that conditions that facilitate retention, including practicing retrieval, are often *more* difficult during initial acquisition (Schmidt & Bjork, 1992, EL: 3). As we note above, for immediate tests, testing is generally *less* effective than restudy, and it is only over the long-term that the benefits of testing emerge. More generally, performance during initial learning is not necessarily a reliable index of long-term learning (Soderstrom & Bjork, 2015, EL: 2).

This principle is counter-intuitive to many learners, in part perhaps because many learners view retrieving information from memory as a process distinct from learning (Karpicke et al., 2009, EL: 5; Kornell & Bjork, 2007, EL: 5; Kornell & Son, 2009, EL: 5; Yan et al., 2014, EL: 5). Intuitively, learners may view practicing retrieval as a way to identify what one does and does not know, but not as way to potentiate learning in and of itself. An analogy is that saving a computer file (“learning”) and opening a file (“retrieval”) are distinct, independent processes. However, the human brain does not operate exactly like a computer, and this naive “storehouse” metaphor is inconsistent with another broad-standing principle of memory (Karpicke, 2012, EL: 6): Retrieval is in fact a potent *modifier* of memory (Anderson et al., 1994, EL: 4) such that each retrieval event itself alters the state of the memory system by making some information more accessible to future retrieval. Psychological scientists have noted the similarity of this phenomenon to the observer effect in physics, where the mere act of observing a particle can alter its condition; similarly, the mere act of retrieving a memory alters it as well (Roediger & Karpicke, 2006b; Spellman & Bjork, 1992).

More recently, researchers have investigated the cognitive mechanisms of testing in particular. One reason that testing may benefit retention is that it increases the number of ways that people can bring to mind the to-be-remembered information (e.g., Bjork, 1975, EL: 3; McDaniel & Masson, 1985, EL: 3; Pyc & Rawson, 2010, EL: 4; Rowland & DeLosh, 2014, EL: 3). For example, it may promote the development of *mediators* between the retrieval environment and the to-be-retrieved material (Pyc & Rawson, 2010, EL: 4). That is, given the need to remember the stages of mitosis (the environment or cue), one might remember *PMAT* (the mediator) in order to retrieve *protophase, metaphase, anaphase, telophase* (the to-be-retrieved targets). More generally, retrieval practice may lead learners to *elaborate* on the target material by bringing to mind additional related information (Carpenter, 2009, EL: 4), which is generally an effective learning technique (Anderson & Reder, 1979, EL: 3). Another, possibly overlapping mechanism may be that retrieval practice enhances the distinctiveness of individual learning episodes (Kuo & Hirshman, 1997, EL: 3; Lehman et al., 2014, EL: 4; Peterson & Mulligan, 2013, EL: 3). For example, the life cycle of the malaria parasite comprises multiple stages, including *sporozoites* and *merozoites*, which learners can easily confuse; however, practice retrieving them from memory makes them more distinct.

Although there remains work to be done to specify the exact cognitive mechanism(s) that underlie the testing effect, the extant literature already supports at least one theoretical conclusion: The testing effect is not an isolated phenomenon. Rather, it follows from broad principles of memory and cognition (transfer-appropriate processing, desirable difficulty, retrieval as a modifier of memory) and can take effect through general cognitive mechanisms (elaboration, distinctiveness, mediators). Because the testing effect is linked to general psychological principles, it is likely to be applicable across a variety of domains and populations, including retention of medical expertise. Nevertheless, the principle of transfer-appropriate processing also implies that testing and retrieval practice will be *most* beneficial when it closely resembles the desired outcome. For instance, retrieval practice with basic factual knowledge alone is less likely to have an impact on clinical behaviors. Rather, assessments will contribute more to learning if they better match the environments physicians encounter in their practice—for instance, by incorporating simulated diagnosis or treatment scenarios.

## Future directions

Although there is robust evidence for the testing effect in general and for several key moderators, we highlight three open questions particularly relevant to the optimal use of using testing in the context of longitudinal assessment of physicians’ medical expertise.

### Use and degree of interleaving

Although meta-analytic evidence indicates learning benefits from interleaving concepts, these studies have employed a variety of learning activities, not only retrieval practice (but see Dobson, 2011, EL: 4, for an example employing retrieval practice). It would be valuable to confirm that the learning benefits of interleaving obtain specifically in the case of testing. Further, most classroom and laboratory studies comparing interleaved and blocked schedules have used a relatively small number of categories or concepts (e.g., four different types of mathematical solids). However, continuing certification program assessments contain many more concepts. Given the hypothesis that interleaving promotes learning by facilitating contrast between confusable concepts, intermixing *all* concepts on continuing certification program assessments may not be optimal because related concepts are unlikely to be adjacent. Indeed, some recent studies (Abel et al., 2021: EL 3; Yan et al., 2021: EL 4) suggest that, in more complex domains, an intermediate rather than maximal degree of interleaving may be optimal precisely because it better facilitates such discriminative contrast. However, this evidence is still early. Thus, we propose comparing the learning benefits of a fully random intermixing of topics versus an order constructed so that potentially confusable topics appear in close proximity. We hypothesize this latter schedule would yield better long-term learning.

### Type of explanation in feedback

Assessments often provide explanations of the correct answer when providing feedback; however, we reviewed evidence that such explanations do not necessarily benefit the learner beyond simply receiving the correct response. A study that manipulates the type of explanations provided during feedback may offer insight into how to improve feedback. One possible design would be to compare later learning outcomes given (a) feedback that uses concrete examples to illustrate a point in addition to providing a technical explanation of how the item should be answered versus (b) only an explanation, but no illustrative example.

### Presence of citations during feedback

Assessments often provide citations alongside evidence for a claim. Some pertinent questions, then, are whether citations benefit learners during feedback, and if so, why. One possibility is that merely having citations builds confidence in the evidence. Another possibility is that the citations are only helpful if physicians actually read the reference. If the testing interface allowed for users to save and/or follow references, log data could be collected to measure these behaviors. The extent to which users engaged with references could be used to predict future performance and provided insight into the value of citations within tests.

## Summary and conclusion

The benefits of testing for learning have been known for over a hundred years and are supported across many domains by a robust literature. The act of retrieving information from one’s memory enhances subsequent retention and results in better learning than restudy, concept mapping, and many other educational techniques.

Here, we considered the relevance of this testing effect for the retention of medical expertise in light of the fact that medical professionals often take periodic tests or assessments as part of their career. For instance, to maintain certification by one of the Member Boards of the American Board of Medical Specialties, physicians in the USA must participate in periodic Maintenance of Certification assessments. However, the principles we have outlined would apply to other professions within the health science, such as nurses or dentists, as well.

The robust evidence for the testing effect implies that such longitudinal assessments can be learning opportunities (“assessment for learning”) as well as summative assessments of a physician’s cognitive skills (“assessment of learning”). A critical goal for any longitudinal assessment program is that the benefits of testing extend beyond future tests and include performance-related outcomes in a medical practice. Fortunately, the reviewed literature indicates that being on some tested information can indeed also improve retention for different, but related information.

The testing effect also generalizes across types of knowledge and tests. A variety of test formats (e.g., short-answer, multiple-choice, etc.) have all been shown to benefit from testing. For this reason, the specific format a test item uses is likely of less importance than the presentational quality of the question (e.g., clarity, readability, and veracity of text). Further, despite the presence of some controversy as to whether the benefits of testing are limited to simple knowledge types (e.g., rote memorization of facts), evidence exists to support improvement in more complex tasks (e.g., problem-solving, clinical skills) as well.

Lastly, although there are some conflicting findings, the testing effect broadly seems to generalize across learners with a range of prior knowledge or cognitive abilities.

The benefits of testing can be further strengthened by leveraging several important moderator variables. First, the positive effects of testing can be reinforced by increasing the retention interval length. Although it is challenging to determine exactly when a subsequent test should occur, given that clinicians are expected to retain their knowledge over the course of an entire career (i.e., several decades), longer retention intervals should be prioritized over shorter intervals. Second, placing gaps between testing sessions themselves maximizes learning outcomes (i.e., spaced repetition). Having tests distributed over time, versus in a contiguous block, should be a key feature to any longitudinal assessment program. Third, switching topics from item to item (interleaving) is likely to be more beneficial than many questions about one topic in a row (blocking). Interleaving may be especially beneficial for easily confused topics, so we suggest using interleaving to bolster cognitive skills and knowledge for targeted areas within medicine (e.g., when two distinct conditions share similar symptoms). Fourth, multiple tests can further boost learning beyond the baseline benefits of a single test, though with diminishing returns. Fifth, ending an assessment “on a high note” with a few relatively easy problems may increase learners’ willingness to engage in future testing by capitalizing on the peak-end rule.

One particularly important moderator is feedback, which enhances the learning benefits of testing and is recommended for any longitudinal assessment framework. Feedback can allay concerns over errors generated during a test, and it is especially important when learners respond wrongly (although feedback also allows learners to improve when they decline to respond). An unsuccessful retrieval attempt followed by feedback is more beneficial than simply reading the correct information without attempting retrieval. In instances where the learner is correct, but has low confidence in their response (e.g., a “lucky guess”), feedback increases the likelihood that the correct response will be later remembered. Further, because corrective feedback allows learners to learn from even difficult tests, learners can be presented with more challenging and demanding tests. In providing feedback to a learner, explanations for correct/incorrect responses have not been reliably shown to aid learning beyond simply providing the correct answers; however, the use of examples during feedback may be useful and is worth further investigation. Citations for sources of information and reference materials may also be beneficial. There remain open questions about *when* learners should receive feedback; we found evidence that delayed feedback may be superior to immediate feedback, but due to sparse evidence in applied domains, we believe this should be tested within medicine.

A final benefit to a longitudinal assessment program is that guiding practitioners to experience the learning benefits of testing, and highlighting these benefits, may lead them to adopt more effective study and learning habits on their own.

Despite robust evidence for the testing effect in general, relatively limited work has examined the efficacy of retrieval practice in physicians, and more rigorous scientific work is needed. The few studies that have been done often involved designs that limit causal attribution (e.g., cross-sectional, self-report, or correlational methods), although a few well-controlled studies do exist (e.g., Larsen et al., 2009). Further, only a relatively small subset of studies in the medical domain have included participants other than medical students or residents. Given the growing emphasis on evidence-based studying practices, more research should be done to assess its efficacy in medicine. Nevertheless, despite these valid limitations, basic-science approaches provide a plethora of evidence that testing should benefit cognitive skills in the domain of medicine. By practicing retrieving information from our memory, we strengthen our memories and increase our knowledge.

## Data Availability

Not applicable.

## Footnotes

1 We thank an anonymous reviewer for suggesting this possibility.
